# Supplementary material for: RNA-dependent chromatin association of transcription elongation factors and Pol II CTD kinases
Source: eLife. 2017 May 24;6:e25637. doi: 10.7554/eLife.25637 (PMC5457138; doi:10.7554/eLife.25637)
Supplement: Supplementary file 1. — YER: Heterochromatic region on chromosome V. Sequences of primer pairs used for strain generation. DOI: http://dx.doi.org/10.7554/eLife.25637.017 [file elife-25637-supp1.docx]

Sequences of primer pairs used for ChIP-qPCR. *YER*: Heterochromatic region on chromosome V.

| **Gene** | Sequence (5'-3') fwd primer | Sequence (5'-3') rev primer |
| --- | --- | --- |
| ***ADH1* (1)** | AGCCGCTCACATTCCTCAAG | ACGGTGATACCAGCACACAAG |
| ***ILV5* (2)** | CTATCAAGCCATTGTTGACC | CTTGAAGACTGGGGAGAAAC |
| ***ILV5* (3)** | ACACCATCAGAAACATGGAA | TGGTTTTCTGGTCTCAACTTT |
| ***PDC1* (4)** | TGTTCGAAAGATTAAAGCAAGTCA | TTGAAGTCACCTGGCAAACC |
| ***PDC1* (5)** | CACTTTCCCAGGTGTCCAAA | CTTAGCGGCGTCAGCAATAG |
| ***PMA1* (6)** | TGACTGATACATCATCCTCTT | TTGGCTGATGAGCTGAAACAGAA |
| ***PMA1* (7)** | GGGTTCCGTGCTTTAGGTGT | CACCCAAGATTTCCCAGTGA |
| ***YER*** | TGCGTACAAAAAGTGTCAAGAGATT | ATGCGCAAGAAGGTGCCTAT |

Sequences of primer pairs used for strain generation.

| **Strain** | Sequence (5'-3') fwd primer | Sequence (5'-3') rev primer |
| --- | --- | --- |
| **ΔRRM-Set1-TAP** | CTTATTTGTTGAATCTTTATAAGAGGTCTCTGCGTTTAGAGAATGAAGAGACAACAGATTGCATCTAAAATTGC | GATATGTTAAATCAGGAAGCTCCAAACAAATCAATGTATCATCGTCGATGAATTCGAGCTCGTTTAAACT |
| **ΔPaf1 Set1-TAP** | GACAGAAATGTATTCAGTACAATAGAACAGTGCTCATAATAGTATAACCGCTAGGGATAACAGGGTA | CTACAGGTTTAAAATCAATCTCCCTTCACTTCTCAATATTCTACGACAGCAGTATAGCGACCA |
